# Increased intratumoral fluorothymidine uptake levels following multikinase inhibitor sorafenib treatment in a human renal cell carcinoma xenograft model

**DOI:** 10.3892/ol.2013.1459

**Published:** 2013-07-12

**Authors:** MASAHIRO MURAKAMI, SONGJI ZHAO, YAN ZHAO, WENWEN YU, CHOWDHURY NUSRAT FATEMA, KEN-ICHI NISHIJIMA, MASAHIRO YAMASAKI, MITSUYOSHI TAKIGUCHI, NAGARA TAMAKI, YUJI KUGE

**Affiliations:** 1 Laboratory of Veterinary Internal Medicine, Graduate School of Veterinary Medicine, Hokkaido University, Sapporo, Hokkaido 060-0818, Japan; 2 Department of Tracer Kinetics and Bioanalysis, Graduate School of Medicine, Hokkaido University, Sapporo, Hokkaido 060-8638, Japan; 3 Department of Nuclear Medicine, Graduate School of Medicine, Hokkaido University, Sapporo, Hokkaido 060-8638, Japan; 4 Oral Diagnosis and Medicine Unit, Department of Oral Pathobiological Science, Graduate School of Dental Medicine, Hokkaido University, Sapporo, Hokkaido 060-8586, Japan; 5 Central Institute of Isotope Science, Hokkaido University, Sapporo, Hokkaido 060-0815, Japan

**Keywords:** fluorothymidine, positron emission tomography, tumor proliferation, anti-angiogenic therapy, Ki-67 labeling index

## Abstract

An early identification of the tumor response to sorafenib treatment is indispensable for selecting optimal personalized treatment strategies. However, at present, no reliable predictors are clinically available. ^18^F-fluorothymidine (^18^F-FLT) positron emission tomography (PET) is used to assess tumor proliferation, since the FLT uptake level reflects thymidine kinase-1 (TK-1) activity. Thus, the present study determined whether FLT was able to evaluate the early tumor response to sorafenib treatment in a human renal cell carcinoma (RCC; A498) xenograft in comparison with the tumor proliferation marker, Ki-67. Mice bearing A498 tumors were assigned to the control and sorafenib-treated groups and the tumor volume was measured every day. [Methyl-3H(N)]-3′-fluoro-3′-deoxythymidine (^3^H-FLT) was injected 2 h prior to the sacrifice of the mice on days three and seven following the treatment. ^3^H-FLT autoradiography (ARG) and Ki-67 immunohistochemistry (IHC) were performed using adjacent tumor sections. In the visual assessment, the intratumoral ^3^H-FLT uptake level diffusely increased following the treatment, while no significant changes were observed in Ki-67 IHC. The intratumoral ^3^H-FLT uptake levels significantly increased by 2.7- and 2.6-fold on days three and seven following the treatment, while the tumor volume and Ki-67 index did not significantly change. Thus, an increased FLT uptake level was demonstrated following the treatment, which may indicate the suppression of thymidylate synthase (TS) and the compensatory upregulation of TK-1 activity by sorafenib.

## Introduction

The treatment options that are available for metastatic renal cell carcinoma (RCC) are limited due to an inherent tumor resistance to chemotherapy and radiotherapy (1). Therefore, as a new treatment strategy, molecular targeting therapies for metastatic RCC have been investigated (2). As a result of the hypervascularity in RCC, the majority of the Food and Drug Administration-approved molecular targeting therapies are anti-angiogenic therapies, which block the signals that are triggered by angiogenic growth factors, including vascular endothelial growth factor (VEGF) and platelet-derived growth factor (PDGF). Sorafenib is an anti-angiogenic agent that inhibits elements of the angiogenesis pathway, including the VEGF receptor (VEGFR) and the PDGF receptor (PDGFR). Sorafenib also inhibits certain processes of tumor proliferation, including the Raf/MEK/ERK pathway, as it is a multikinase inhibitor (3). Since the main mechanism of therapeutic action is anti-angiogenesis, which shows no direct cytotoxicity, the therapeutic effect is difficult to evaluate using tumor volume measurement methods, including the Response Evaluation Criteria In Solid Tumors (4). Furthermore, the early identification of the tumor response to sorafenib treatment is indispensable for selecting optimal personalized treatment strategies, but at present, no reliable predictors are clinically available. The mechanisms of action for sorafenib involve anti-angiogenesis and the inhibition of tumor proliferation. Tumor proliferation is a useful marker to evaluate the therapeutic effect and prognosis following therapy in clinical oncology (5,6). Therefore, the evaluation of tumor proliferation following sorafenib treatment may reflect the response of the tumor to the treatment. Histopathological analysis using the Ki-67 labeling index is a gold standard for the evaluation of tumor proliferation (7). However, the Ki-67 labeling index may only be used to evaluate tumor proliferation in biopsy samples or excised tumor tissues. Thus, a non-invasive method to evaluate tumor proliferation is required. ^18^F-fluorothymidine (^18^F-FLT) positron emission tomography (PET), which reflects thymidine kinase-1 (TK-1) activity, is a non-invasive method for detecting tumor proliferation (8,9). Certain studies have demonstrated the attenuation of tumor proliferation following radiotherapy or chemotherapy detected by FLT PET (10–15). However, the changes in intratumoral FLT distribution following sorafenib treatment are yet to be clarified. Thus, the present study assessed whether FLT may be used to evaluate the early tumor response to sorafenib treatment in an RCC xenograft, and compared the results with those from an assessment using the tumor proliferation marker, Ki-67.

## Materials and methods

### Tumor xenograft model and sorafenib treatment

**Figure 1:**
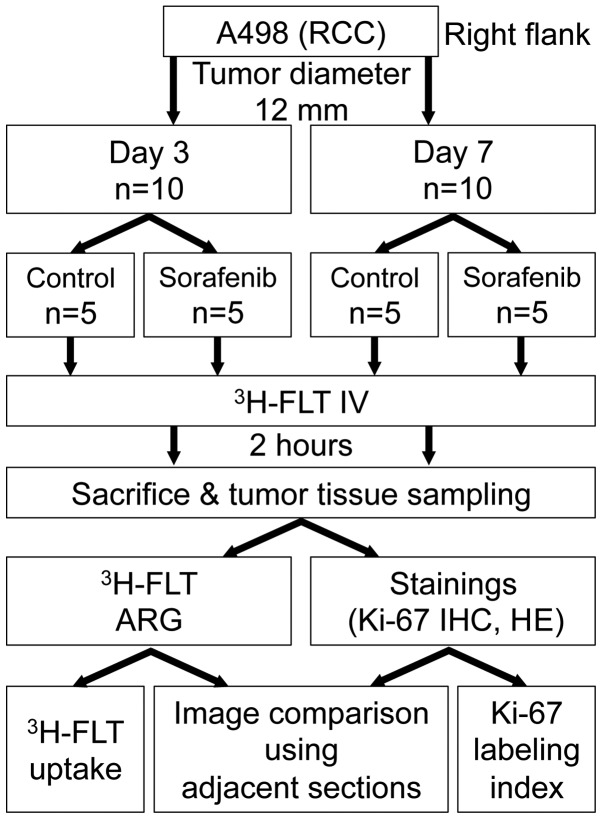
Experimental procedures of the present study. RCC, renal cell carcinoma; Control, control group; Sorafenib, sorafenib-treated group; ^3^H-FLT, [methyl-3H(N)]-3′-fluoro-3′-deoxythymidine; ARG, autoradiography; IHC; immunohistochemistry; HE, hematoxylin and eosin.

Nine-week-old male BALB/c athymic nude mice (Japan SLC, Inc., Hamamatsu, Japan) were used in the present study. Approval for the study was obtained from the Laboratory Animal Care and Use Committee of Hokkaido University (Sapporo, Hokkaido, Japan). A human RCC xenograft model was established using the human clear cell RCC (A498) cell line (European Collection of Cell Cultures, Salisbury, UK), which is a von Hippel-Lindau (VHL) mutant. The A498 cells were maintained in RPMI-1640 medium (Invitrogen Life Technologies, Inc., Carlsbad, CA, USA), supplemented with 10% fetal bovine serum, penicillin-streptomycin and 0.03% glutamine, and incubated in an atmosphere of 5% CO_2_ and 95% air at 37°C. The A498 cells (1×10^7^ cells/0.1 ml) were subcutaneously inoculated into the right flank of each mouse. When the tumors grew to 12 mm in diameter, the mice were randomly assigned to two groups, the day three and day seven groups (n=10 per group). The mice were then further assigned to the control and sorafenib-treated subgroups within each group (n=5 per subgroup; Fig. 1). In the sorafenib-treated groups, sorafenib (80 mg/kg; Nexavar, Bayer Pharmaceuticals Corporation, West Haven, CT, USA), in a Cremophor EL (Sigma, St. Louis, MO, USA) ethanol (Pharmaco Products, Brookfield, CT, USA) and water solution (12.5:12.5:75) was administered daily by oral gavage. The Cremophor EL/ethanol/water solution was administered as the vehicle in the control groups. A tumor growth curve was derived from the day seven group. The tumor size was measured using a caliper every day from the first day of treatment, and the tumor volume was calculated using the following formula: π/6 × larger diameter × (smaller diameter)^2^. The change in the tumor volume was calculated using the following formula: (tumor volume of each day) − (tumor volume of day 0).

### [Methyl-^3^H(N)]-3′-fluoro-3′-deoxythymidine (^3^H-FLT) autoradiography (ARG) Ki-67 immunohistochemistry (IHC) and hematoxylin and eosin (HE) staining

^3^H-FLT (specific activity, 74–370 GBq/mmol) was purchased from Moravek Biochemicals Inc. (Brea, CA, USA). Mice were injected with 0.185 MBq ^3^H-FLT into the tail vein. At two hours post-^3^H-FLT injection, the mice were sacrificed and the tumors and muscles were immediately excised. Each excised tumor tissue was then sectioned into 2–3-mm thick slices to maximize the division surface, then embedded in Tissue-Tek medium (Sakura Finetechnical Co., Ltd., Tokyo, Japan) with the calf muscle and frozen in isopentane/dry ice. An adjacent 10-μm cryosection and two adjacent 5-μm cryosections were prepared with a CM3050-Cryostat (Leica Microsystems, Tokyo, Japan) and used for ARG, IHC and HE staining, respectively. The 10-μm sections were placed in a phosphor image plate cassette with a set of calibrated standards (16), and ARG exposure was performed for four weeks to detect the distribution of ^3^H-FLT. The ARG images were analyzed using a computerized imaging analysis system (FLA 7000 Bio-Imaging Analyzer; Fuji Photo Film Co., Ltd., Minato-ku, Tokyo, Japan). An adjacent 5-μm section was immunohistochemically stained for Ki-67 to assess the tumor proliferation. Briefly, following rehydration and antigen retrieval, endogenous peroxidase activity was blocked using methanol containing 0.3% hydrogen peroxide. Thereafter, the sections were incubated with a monoclonal rabbit anti-human Ki-67 antibody (Clone SP6; Thermo Fisher Scientific, Waltham, MA, USA). The bound antibodies were visualized using the avidin/biotin conjugate immunoperoxidase procedure with a Histofine SAB-PO kit (Nichirei Biosciences Inc., Tokyo, Japan) and 3,3′-diaminobenzidine tetrahydrochloride. The slides were counterstained using Mayer’s hematoxylin solution (Wako, Osaka, Japan). The IHC images of the tumor sections that were stained for Ki-67 were captured under a microscope (Biozero BZ-8000; Keyence Co., Osaka, Japan), and converted to black and white images using Image J (National Institutes of Health, Bethesda, MD, USA). For the assessment of the distribution pattern, the ARG images of ^3^H-FLT and the images of the adjacent sections that were stained for Ki-67 by IHC were visually compared. The remaining adjacent 5-μm sections were stained with HE to determine the regions of interest (ROIs) for the quantitative analysis of ^3^H-FLT using the ARG images.

### Quantitative analysis of ^3^H-FLT ARG image and Ki-67 IHC

To quantitatively evaluate ^3^H-FLT radioactivity, the ROIs were placed to cover the entire tumor tissue on each ARG image with reference to the HE-stained sections. The radioactivity in each ROI was calculated using the activity of the standards and expressed as a percentage of the injected dose (ID) per gram of tissue following normalization to the animal’s body weight [(%ID/g) × kg] (16).

For the quantitative analysis of tumor proliferation, the Ki-67 labeling index, i.e. a percentage of the number of Ki-67-positive nuclei to the total number of nuclei, was used. To obtain the Ki-67 labeling index, the numbers of Ki-67-positive nuclei and nuclei that were stained using Mayer’s hematoxylin (all nuclei) were counted under a microscope field (x400 objective magnification, 0.644 mm^2^ per field) using Image J. A total of 10 fields per section were randomly analyzed, excluding the peripheral connective tissue and central necrotic tissue.

### Statistical analyses

**Figure 2:**
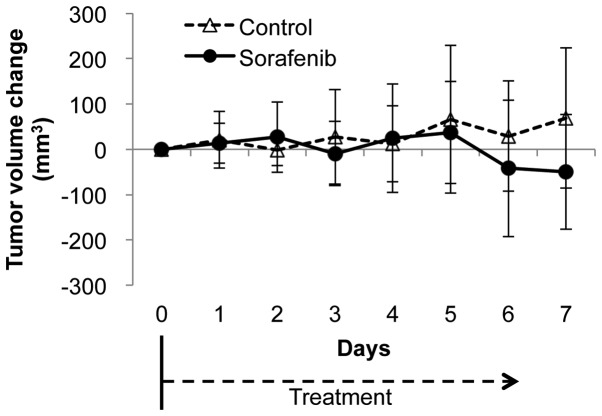
Changes in tumor volume following treatment with the vehicle or sorafenib. Dotted arrow, treatment period. Control, control group; Sorafenib, sorafenib-treated group..

All statistical analyses were carried out using StatView version 5.0 (SAS Institute Inc., Cary, NC, USA). All values are expressed as mean ± SD. One-way repeated measures analysis of variance (ANOVA) was used to assess the significant differences in the trends of the tumor volume changes between the control and treatment groups (Fig. 2). In the evaluation of ^3^H-FLT distribution by ARG and the Ki-67 labeling index (Fig. 4), the Mann-Whitney U test was used to assess the significant differences between the control and treatment groups on days three and seven. P<0.05 was considered to indicate a statistically significant difference.

## Results

### Tumor volume change

The changes in the tumor volume are shown in Fig. 2. No statistically significant differences were observed between the control and sorafenib-treated groups during the study period until day seven (P=0.59).

### Image comparison between ^3^H-FLT ARG and Ki-67 IHC

**Figure 3:**
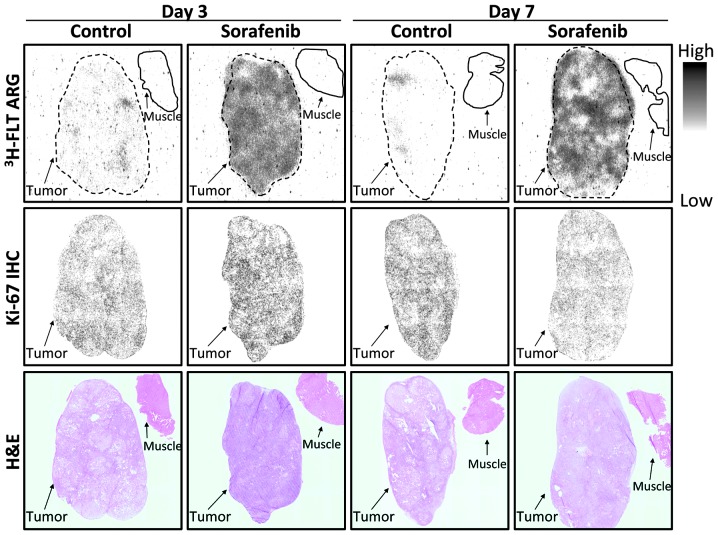
Representative images of ^3^H-FLT ARG, immunohistochemical stainings of Ki-67 and HE stainings on days three and seven following treatment with the vehicle or sorafenib. The dotted line represents the tumor outline. The solid line represents the muscle outline. ^3^H-FLT, [methyl-3H(N)]-3′-fluoro-3-′deoxythymidine; ARG, autoradiography; Control, control group; Sorafenib, sorafenib-treated group; HE, hematoxylin and eosin; IHC, immunohistochemistry.

Fig. 3 shows representative images of ^3^H-FLT ARG, Ki-67 IHC and HE staining. In the control groups, the ^3^H-FLT ARG images revealed low levels of intratumoral ^3^H-FLT distribution on days three and seven, which were similar to those observed in the muscle. The intratumoral ^3^H-FLT uptake level was diffuse and markedly increased in the sorafenib-treated groups compared with the control groups. There were no significant differences in the level of intratumoral ^3^H-FLT distribution between days three and seven, whereas a more heterogeneous intratumoral ^3^H-FLT distribution was observed on day seven compared with day three in the sorafenib-treated group. The distribution profiles of the Ki-67-positive nuclei on days three and seven in the sorafenib-treated groups were visually similar to those in the control groups. There were no significant differences in the distribution level of Ki-67-positive nuclei between days three and seven in the control and sorafenib-treated groups.

### Quantitative analysis of ^3^H-FLT ARG image and Ki-67 IHC

**Figure 4:**
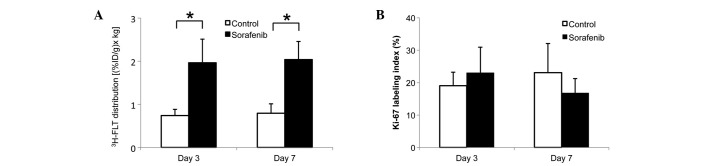
Quantitative analysis of intratumoral (A) ^3^H-FLT distribution and (B) Ki-67 labeling index on days three and seven following treatment with vehicle or sorafenib. ^3^H-FLT, [methyl-3H(N)]-3′-fluoro-3-′deoxythymidine; ID, injected dose; Control, control group; Sorafenib, sorafenib-treated group. ^*^P<0.01.

Fig. 4A shows the quantitative evaluation of the intratumoral ^3^H-FLT distribution on days three and seven following treatment with the vehicle or sorafenib. The levels of ^3^H-FLT uptake in the tumors were 0.74±0.15 and 1.96±0.54 [(%ID/g) × kg] on day three (P<0.01) and 0.80±0.21 and 2.04±0.42 [(%ID/g) × kg] on day seven (P<0.01) in the control and sorafenib-treated groups, respectively. The intratumoral ^3^H-FLT uptake levels significantly increased by 2.7- and 2.6-fold on days three and seven following the treatment with sorafenib, respectively, compared with the control groups.

Fig. 4B shows the quantitative evaluation of Ki-67 IHC on days three and seven following the treatment with the vehicle or sorafenib. The Ki-67 labeling indices in the tumors were 19.1±4.2 and 23.0±7.9% on day three and 23.1±9.0 and 17.1±3.8% on day seven in the control and sorafenib-treated groups, respectively. On days three and seven following the treatment with sorafenib, the Ki-67 labeling indices were not significantly different from those of the control groups.

## Discussion

A major finding of the present study is that the level of ^3^H-FLT uptake diffusely and significantly increased following the treatment with sorafenib compared with the control groups (Figs. 3 and 4A), even though the Ki-67-positive cell distribution, Ki-67 labeling index and tumor volume did not display significant changes between the sorafenib-treated and control groups (Figs. 2, 3 and 4B). At first, the FLT uptake level was expected to decrease in concert with the suppression of tumor proliferation (Ki-67 labeling index decrease) by the sorafenib treatment. However, the present findings unexpectedly revealed that the FLT uptake level in the RCC xenograft significantly increased following the sorafenib treatment without significant changes in the tumor proliferation marker level (Ki-67 labeling index) or the tumor volume.

Sorafenib is a multikinase inhibitor whose action mechanisms include the inhibition of the tumor proliferative signaling pathway (17). Therefore, the proliferation marker and ^3^H-FLT uptake levels were expected to decrease following sorafenib treatment in an A498 xenograft. FLT is generally used as a tumor proliferation marker in clinical oncology (8,9,18). Numerous studies have demonstrated a decrease in the FLT uptake level following conventional chemotherapy and, in certain reports, subsequent to molecular targeted therapy (19). However, in the present study, the ^3^H-FLT uptake level increased dramatically following the sorafenib treatment in an A498 xenograft. To the best of our knowledge, no study has shown an increase in FLT uptake level during molecular targeted therapy. Only one study has suggested an increase in FLT uptake level following the cessation of several days of treatment using the multikinase inhibitor sunitinib malate in a clinical setting (20).

**Figure 5:**
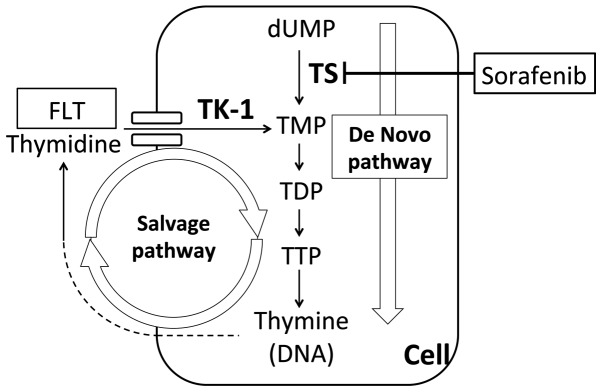
Schematic pathways of the thymidine supply for DNA synthesis. dUMP, deoxyuridine monophosphate; TMP, thymidine monophosphate; TDP, thymidine diphosphate; TTP, thymidine triphosphate; TS, thymidate synthase; FLT, fluorothymidine; TK-1, thymidine kinase-1.

In addition, the increase in FLT uptake level was inconsistent with the absence of significant changes in the Ki-67 labeling index in the present study. Recent studies have revealed the discordance between the level of FLT uptake and other tumor proliferation markers (21,22). One of the potential causes of the increase in FLT uptake level without an increase in the level of proliferation markers is the upregulation of TK-1 activity that arises from the inhibition of thymidylate synthase (TS). Several studies have shown that the FLT uptake level reflects TS inhibition by fluorouracil (5-FU) treatment independent of the tumor proliferation changes (23,24). A schematic diagram of the thymidine supply for DNA synthesis is shown in Fig. 5. There are two pathways of thymidine supply for DNA synthesis, the *de novo* pathway and the salvage pathway. TS and TK-1 are critical enzymes in the *de novo* and salvage pathways, respectively. When the *de novo* pathway is suppressed, the salvage pathway is compensatorily upregulated to maintain a certain level of thymidine supply (24,25). Thus, TS inhibition or suppression increases TK-1 activity and FLT uptake (24).

With regard to the effect of sorafenib on the thymidine supply pathways, only one study has suggested the suppression of TS in RCC cells following sorafenib treatment (26). The increase in the FLT uptake level following sorafenib treatment in the present study may have been caused by the TS suppressive effect of sorafenib, which upregulates the thymidine salvage pathway. The present study strongly indicated the importance of determining whether the treatment affects the activity of TS when evaluating the treatment response by FLT PET.

In addition to the fact that the FLT uptake level directly reflects TK1 activity but not tumor proliferation, the technical aspects, including the difference in the samples used for the evaluation of FLT uptake and Ki-67, should be considered as another reason for the inconsistency between the level of ^3^H-FLT uptake and the Ki-67 labeling index. However, in the present study, tumor-adjacent sections were used for ^3^H-FLT ARG and Ki-67 IHC image comparison, which enabled the comparison of the distributions of FLT and Ki-67-positive cells at a microscopic level. Additionally, in the present ARG experiments, ^3^H-FLT was used instead of ^18^F-FLT, even though ^18^F-FLT has been extensively used to determine FLT distribution. The use of ^3^H-FLT produced clearer images and provided more precise information on the FLT distribution than that of ^18^F-FLT, owing to the shorter radiation range of ^3^H.

In conclusion, the intratumoral ^3^H-FLT distribution was significantly increased following sorafenib treatment in a human RCC xenograft, even though the tumor proliferation marker Ki-67 labeling index and the tumor volume did not significantly change. Thus, an increased FLT uptake level following treatment may indicate the suppression of TS and a compensatory upregulation of TK-1 activity. Further studies are required to clarify the mechanisms underlying the increased FLT uptake following sorafenib treatment, which may lead to the application of FLT PET for monitoring the treatment effects.

## Abbreviations

ARG: autoradiography
FLT: fluorothymidine
HE: hematoxylin and eosin
IHC: immunohistochemistry
PDGF: platelet-derived growth factor
PDGFR: platelet-derived growth factor receptor
RCC: renal cell carcinoma
ROI: region of interest
TS: thymidylate synthase
TK-1: thymidine kinase-1
VEGF: vascular endothelial growth factor
VEGFR: vascular endothelial growth factor receptor
VHL: von Hippel-Lindau
